# Whole-lesion histogram analysis of multiple diffusion metrics for differentiating lung cancer from inflammatory lesions

**DOI:** 10.3389/fonc.2022.1082454

**Published:** 2023-01-18

**Authors:** Jiaxin Li, Baolin Wu, Zhun Huang, Yixiang Zhao, Sen Zhao, Shuaikang Guo, Shufei Xu, Xiaolei Wang, Tiantian Tian, Zhixue Wang, Jun Zhou

**Affiliations:** ^1^ Department of Radiology, The First Affiliated Hospital of Henan University, Kaifeng, China; ^2^ Huaxi MR Research Center (HMRRC), Department of Radiology, West China Hospital of Sichuan University, Chengdu, China; ^3^ Department of Radiology, Henan Provincial People’s Hospital, Zhengzhou, China; ^4^ Department of Critical Care Medicine, The First Affiliated Hospital of Henan University, Kaifeng, China; ^5^ Department of Radiology, Huaihe Hospital of Henan University, Kaifeng, China; ^6^ Interventional Diagnostic and Therapeutic Center, Zhongnan Hospital of Wuhan University, Wuhan, China

**Keywords:** diffusion-weighted imaging, intravoxel incoherent motion, diffusion kurtosis imaging, magnetic resonance imaging, lung lesions, histogram analysis

## Abstract

**Background:**

Whole-lesion histogram analysis can provide comprehensive assessment of tissues by calculating additional quantitative metrics such as skewness and kurtosis; however, few studies have evaluated its value in the differential diagnosis of lung lesions.

**Purpose:**

To compare the diagnostic performance of conventional diffusion-weighted imaging (DWI), intravoxel incoherent motion (IVIM) magnetic resonance imaging (MRI) and diffusion kurtosis imaging (DKI) in differentiating lung cancer from focal inflammatory lesions, based on whole-lesion volume histogram analysis.

**Methods:**

Fifty-nine patients with solitary pulmonary lesions underwent multiple *b*-values DWIs, which were then postprocessed using mono-exponential, bi-exponential and DKI models. Histogram parameters of the apparent diffusion coefficient (ADC), true diffusivity (*D*), pseudo-diffusion coefficient (D*), and perfusion fraction (*f*), apparent diffusional kurtosis (K_app_) and kurtosis-corrected diffusion coefficient (D_app_) were calculated and compared between the lung cancer and inflammatory lesion groups. Receiver operating characteristic (ROC) curves were constructed to evaluate the diagnostic performance.

**Results:**

The ADC^mean^, ADC^median^, *D* ^mean^ and *D* ^median^ values of lung cancer were significantly lower than those of inflammatory lesions, while the ADC^skewness^, K_app_^mean^, K_app_^median^, K_app_^SD^, K_app_^kurtosis^ and D_app_^skewness^ values of lung cancer were significantly higher than those of inflammatory lesions (all *p* < 0.05). ADC^skewness^ (*p* = 0.019) and *D* ^median^ (*p* = 0.031) were identified as independent predictors of lung cancer. *D* ^median^ showed the best performance for differentiating lung cancer from inflammatory lesions, with an area under the ROC curve of 0.777. Using a *D* ^median^ of 1.091 × 10^-3^ mm^2^/s as the optimal cut-off value, the sensitivity, specificity, positive predictive value and negative predictive value were 69.23%, 85.00%, 90.00% and 58.62%, respectively.

**Conclusions:**

Whole-lesion histogram analysis of DWI, IVIM and DKI parameters is a promising approach for differentiating lung cancer from inflammatory lesions, and *D* ^median^ shows the best performance in the differential diagnosis of solitary pulmonary lesions.

## Introduction

Lung cancer has been the most common cancer in China, and is the leading cause of cancer-related deaths in both China and United States (1). In clinical practice, detection of lung lesions mainly relies on computed tomography (CT) due to its short-time scanning and high resolution of density. As a preferred imaging technique for detecting lung lesions, CT imaging can capture the important features of lesions in terms of morphology, boundary, tissue density and enhancement. However, given that lung cancer and some benign lesions (e.g., inflammatory lesions) usually have overlapped features on CT, it is occasionally difficult to distinguish lung cancer from benign lesions. Although CT-guided transthoracic lung biopsy has been widely used for pathologic diagnosis of lung lesions, this technique is invasive and not suitable for patients with poor lung function. Thus, it is essential to develop non-invasive and valuable tools for the accurate diagnosis of lung lesions. Early diagnosis and differentiation of pulmonary lesions will help to select the optimal therapeutic strategy and avoid some unnecessary treatments.

In recent years, diffusion-weighted imaging (DWI), a non-invasive magnetic resonance imaging (MRI) technique, has shown potential diagnostic value for lung lesions. Previous studies have demonstrated that the apparent diffusion coefficient (ADC), a parameter calculated from DWI data based on a mono-exponential model, can be used to distinguish malignant and benign pulmonary lesions (2, 3), and may also help characterize the subtype of lung cancer (2). However, the ADC value is also affected by tissue microcirculation perfusion. Compared with conventional DWI, intravoxel incoherent motion (IVIM) MR imaging allows separate calculation of diffusion and perfusion parameters based on a bi-exponential model (4). Additionally, another advanced diffusion model, diffusion kurtosis imaging (DKI), has been a new topic of growing interest in radiology and enables characterization of non-Gaussian water diffusion behavior (5). Considering that analyses using mono- and bi-exponential DWI models and DKI model provide information on different aspects of tissue microstructure, it is necessary to explore and compare their roles in the differentiation of solitary pulmonary lesions, and thus helping to select the optimal imaging parameter for lesion diagnosis. In fact, several studies have been conducted to address this issue (3, 6, 7), but did not yield consistent and conclusive results. It should be noted that most of previous studies analyzed the diffusion MR imaging parameters based on single-section (usually selected the slice with the largest diameter of lung lesions) regions of interest (ROIs) rather than whole-lesion volumes. Traditional single-section ROI analysis cannot capture the heterogeneity within the whole lesions, which may lead to subjective bias and possible sampling error of measurement.

Whole-lesion histogram analysis, a method that can provide comprehensive microstructural information of lesions by calculating additional quantitative metrics (such as skewness and kurtosis), has been increasingly applied in cancer imaging researches. This volumetric analysis can address internal lesion heterogeneity, and has better interobserver reproducibility and higher diagnostic accuracy compared with single-section ROI analysis (8, 9). Quantitative whole-lesion histogram analysis has been used to differentiate histological grades of rectal cancer (10) and gastric cancer (11), to distinguish benign and malignant breast lesions (12), and to assess response to combined chemotherapy and radiation therapy (CRT) in patients with rectal cancer (8). However, to our knowledge, studies using whole-lesion histogram analysis of mono- and bi-exponential DWI and DKI for differentiation of lung lesions are limited. Thus, the present study aimed to evaluate the diagnostic performance of conventional ADC, IVIM and DKI metrics in differentiating primary lung cancer from focal inflammatory lesions, by using histogram analysis derived from whole-lesion volumes.

## Materials and methods

### Study population

This prospective single-center study was approved by the Institutional Review Board of The First Affiliated Hospital of Henan University. Written informed consent was obtained from each patient prior to inclusion. From February 2022 to September 2022, consecutive patients who met the following inclusion criteria were recruited: (1) pulmonary solitary lesions detected by initial CT examinations, and the maximum diameter of the lesion was greater than or equal to 15 mm; (2) the pulmonary lesions could be clearly visualized on subsequent MR imaging; (3) the solid component accounted for more than 50% of the whole lesion; and (4) no radiochemotherapy or targeted drug therapy was performed prior to MRI examinations. The exclusion criteria were: (1) CT images showed ground glass foci; (2) poor physical condition that led to intolerance to MRI examinations; (3) contraindication to MR scanning, such as claustrophobia and placement of artificial cardiac pacemaker; and (4) poor MR imaging quality that made it impossible to perform data post-processing.

CT-guided transthoracic needle biopsy or surgery was performed within 10 days after MRI examinations. Diagnosis of primary lung cancer was confirmed by pathological results, while diagnosis of inflammatory lesions was confirmed by either pathological results or radiological follow-up more than one month after anti-inflammatory treatment (manifested as the disappearance or obvious regression of the lesions).

Seventy-four consecutive patients were included in this study, and 15 patients were excluded due to the following reasons: (1) other types of space-occupying lung lesions, including low-grade mucoepidermoid carcinoma (n = 1), atypical hyperplasia (n = 1), low-grade intraepithelial neoplasia (n = 1), lymphoma (n = 1), and hamartoma (n = 2); (2) contraindication to MR scanning (n = 4); and (3) poor imaging quality (n = 5). Thus, the MR imaging data of lung lesions in the remaining 59 patients (27 males and 32 females; mean age 57.8 ± 9.9, range from 39 to 79 years) were used for final analyses.

### MRI data acquisition

Within one week after the lesions were detected by CT, all patients underwent MR imaging using a 1.5-T MRI scanner (MAGNETOM Sempra, Siemens Healthcare, Erlangen, Germany) with a 13-channel body coil. First, conventional axial T1-weighted, as well as axial and coronal T2-weighted imaging sequences were acquired. Then, conventional DWI, IVIM and DKI images were obtained using multiple *b*-values. Detailed MR imaging parameters are shown in **Table 1**.

**Table 1:** MR imaging parameters.

Parameter	Axial T1WI	Axial T2WI	Coronal T2WI	Axial DWI	Axial IVIM	Axial DKI
Imaging technique	3D VIBE	BLADE	HASTE	SE-EPI	SE-EPI	SE-EPI
Respiratory compensation	Breath holding	Respiratory-triggered	Breath holding	Free breathing	Free breathing	Free breathing
TR/TE (ms)	2.6/0.92	4000/74	1100/51	5300/67	3000/76	4200/91
FOV (mm^2^)	380 × 285	380 × 380	380 × 380	380 × 310	380 × 310	380 × 310
Matrix	224 × 224	240 × 240	256 × 230	128 × 128	128 × 128	128 × 128
Section thickness (mm)	5	5	5	5	5	5
Section gap (mm)	1	1	1	1	1	1
No. of sections	10	10	30	10	10	10
*b-*values (s/mm^2^)	…	…	…	0 and 800	0, 10, 20, 30, 50, 80, 150, 300, 500, 800, and 1000	0, 500, 1000, 1500, and 2000

T1WI, T1-weighted imaging; T2WI, T2-weighted imaging; DWI, diffusion-weighted imaging; IVIM, intravoxel incoherent motion; DKI, diffusion kurtosis imaging; 3D-VIBE, three-dimensional volumetric interpolated breath-hold examination; HASTE, half-Fourier acquisition single-shot turbo spin echo; SE-EPI, spin-echo echo-planar imaging; TR, repetition time; TE, echo time; FOV, field of view.

### Image analysis

MRI data analysis was performed using the Medical Imaging Interaction Toolkit (MITK) Workbench. Multiple *b*-values DWI data were post-processed using mono-exponential (*b*-values = 0 and 800 s/mm^2^), bi-exponential IVIM (*b*-values = 0, 10, 20, 30, 50, 80, 150, 300, 500, 800, and 1000 s/mm^2^) and DKI (*b*-values = 0, 500, 1000, 1500, and 2000 s/mm^2^) models. Subsequently, the values of the ADC, IVIM parameters [true molecular diffusion coefficient (*D*), pseudo-diffusion coefficient (D*), and perfusion fraction (*f*)] and DKI parameters [apparent diffusional kurtosis (K_app_) and kurtosis-corrected diffusion coefficient (D_app_)] were calculated. The calculation equations and the meanings for ADC, IVIM and DKI parameters have been described in detail in previous studies (3, 13).

For further histogram analyses, ROIs were manually delineated layer by layer on ADC maps by referring to the conventional axial T2-weighted images, and areas with vessels, cavity and necrosis within the lesions were excluded, thus generating a three-dimensional volume of interest (VOI). The ROIs were automatically copied from the ADC maps to the corresponding IVIM and DKI parametric maps. Then, histogram analyses of these quantitative MR parameters were performed based on the information extracted from each voxel within the entire VOI. The calculated histogram parameters included: (1) mean; (2) median; (3) standard deviation (SD); (4) skewness, which measures the degree of histogram asymmetry around the mean; and (5) kurtosis, which measures the histogram sharpness.

Two of the authors (JXL and BLW, radiologists with five and seven years of experience in chest imaging, respectively) who were blinded to the histopathological results independently performed data analysis.

### Statistical analysis

Statistical analyses were performed using SPSS version 21.0 (IBM Corp, Armonk, NY) and MedCalc version 19.2.0 (MedCalc, Mariakerke, Belgium) software. Between-group differences in demographic and clinical data, as well as in all histogram parameters were determined using chi-square test, two-sample *t*-test and Mann-Whitney *U*-test. We calculated the intraclass correlation coefficient (ICC) by using a two-way random-effects model to evaluate the consistency and reliability of the same histogram parameter between the two independent observers (< 0.40, poor agreement; 0.40–0.59, fair agreement; 0.60–0.74, good agreement; and ≥ 0.75, excellent agreement). For histogram metrics showing significant between-group differences, univariate and multivariate logistic regression analyses were performed to determine the independent predictors of lung cancer. Furthermore, we used receiver operating characteristic (ROC) curves to assess the diagnostic performance of each histogram parameter in differentiating lung cancer from inflammatory lesions, and identified the corresponding optimal cut-off value. Then, the area under the ROC curve (AUC), sensitivity, specificity, positive predictive value (PPV) and negative predictive value (NPV) were calculated. The DeLong test (14) was used to compare the AUC values of different histogram metrics, and the Benjamin–Hochberg false discovery rate was used to correct for multiple comparisons (15). A *p* value less than 0.05 was considered to indicate statistical significance.

## Results

### Demographic and clinical characteristics

According to the pathological results or radiological follow-up after anti-inflammatory treatment, the included 59 patients were divided into two groups: the lung cancer group (n = 39) and the inflammatory lesion group (n = 20). The demographic and clinical characteristics of the two patient groups are shown in **Table 2**. There were no significant differences in age, sex, lesion size and lesion location between the lung cancer and inflammatory lesion groups (all *p* > 0.05).

**Table 2:** Demographic and clinical characteristics of the two patient groups.

	Lung cancer (n = 39)	Inflammatory lesions (n = 20)	*p* value
Age (years)	59.2 ± 9.3	55.1 ± 10.8	0.130
Gender, *n* (%)			0.933
Male	18 (46.2%)	9 (45.0%)	
Female	21 (53.8%)	11 (55.0%)	
Lesion size (mm)	28.9 ± 8.0	30.7 ± 7.7	0.431
Lesion location, *n* (%)			0.480
Right upper lobe	15 (38.5%)	8 (40.0%)	
Right middle lobe	1 (2.6%)	2 (10.0%)	
Right lower lobe	8 (20.5%)	4 (20.0%)	
Left upper lobe	10 (25.6%)	2 (10.0%)	
Left lower lobe	5 (12.8%)	4 (20.0%)	
Diagnosis, *n*	Adenocarcinoma, 27; Squamous cell carcinoma, 12	Lung abscess, 10; tuberculosis, 4; pneumonia, 6	

### Interobserver agreement

As show in **Table 3**, all the ADC, *D*, D*, *f*, K_app_ and D_app_ histogram parameters of pulmonary lesions showed excellent or good interobserver agreement, with an ICC range from 0.678 to 0.894.

**Table 3:** Intra-observer agreement on whole-volume histogram parameters.

Parameter	ICCs	95% CI	Parameter	ICCs	95% CI
*ADC histogram*			*f histogram*		
Mean	0.849	(0.750-0.909)	Mean	0.774	(0.626-0.863)
Median	0.838	(0.731-0.903)	Median	0.743	(0.570-0.846)
Standard deviation	0.876	(0.794-0.926)	Standard deviation	0.678	(0.398-0.820)
Skewness	0.799	(0.667-0.879)	Skewness	0.766	(0.611-0.859)
Kurtosis	0.843	(0.740-0.905)	Kurtosis	0.723	(0.542-0.832)
*D histogram*			*K_app_ histogram*		
Mean	0.768	(0.615-0.861)	Mean	0.834	(0.434-0.930)
Median	0.883	(0.768-0.936)	Median	0.842	(0.415-0.936)
Standard deviation	0.834	(0.654-0.912)	Standard deviation	0.871	(0.786-0.922)
Skewness	0.807	(0.680-0.883)	Skewness	0.881	(0.797-0.929)
Kurtosis	0.800	(0.667-0.879)	Kurtosis	0.863	(0.770-0.918)
D* histogram			*D_app_ histogram*		
Mean	0.785	(0.633-0.872)	Mean	0.839	(0.732-0.903)
Median	0.724	(0.535-0.836)	Median	0.875	(0.794-0.925)
Standard deviation	0.745	(0.575-0.846)	Standard deviation	0.801	(0.670-0.880)
Skewness	0.840	(0.734-0.903)	Skewness	0.894	(0.823-0.936)
Kurtosis	0.799	(0.666-0.879)	Kurtosis	0.807	(0.154-0.928)

ICCs, intraclass correlation coefficients; CI, confidence interval.

### Between-group differences in whole-lesion histogram metrics

**Figure 1:**
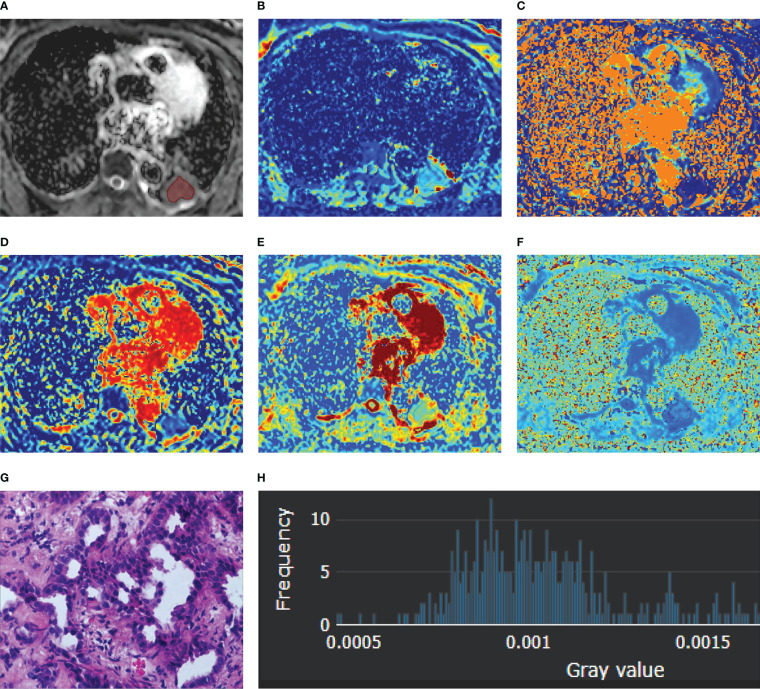
A 56-year-old female diagnosed with adenocarcinoma of the left lower lobe. **(A)** ADC map; **(B)** D map; **(C)** D* map; **(D)** *f* map **(E)** Dapp map; **(F)** Kapp map; **(G)** microscopic image of H&E staining (original magnification, ×200); **(H)** an example diagram of the histogram.

**Figure 2:**
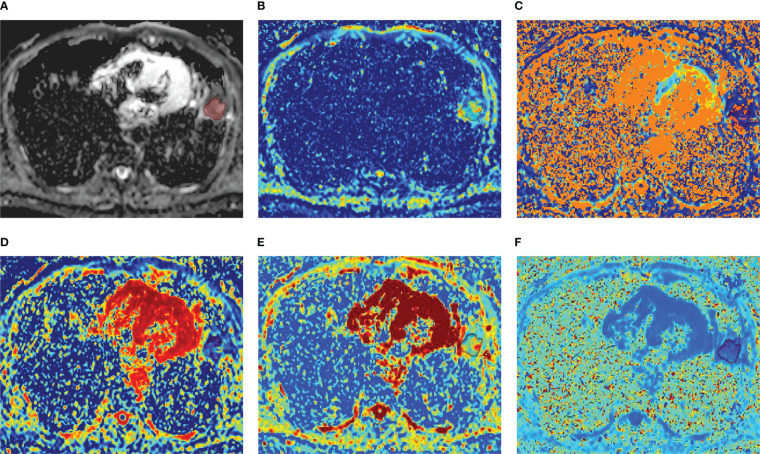
** **A 52-year-old male diagnosed with lung abscess of the left lower lobe. **(A)** ADC map; **(B)** D map; **(C)** D* map; **(D)** f map **(E)** Dapp map; **(F)** Kapp map.

Compared to inflammatory lesions, lung cancer had significantly lower ADC^mean^, ADC^median^, *D* ^mean^ and *D* ^median^ values, and showed significantly higher ADC^skewness^, K_app_^mean^, K_app_^median^, K_app_^SD^, K_app_^kurtosis^ and D_app_^skewness^ values (all *p* < 0.05) (**Table 4**). No significant differences in ADC^SD^, ADC^kurtosis^, *D* ^SD^, *D* ^kurtosis^, *D* ^skewness^, Kapp^skewness^, D_app_^mean^, D_app_^median^, D_app_^SD^ and D_app_^kurtosis^ values were observed between the lung cancer and inflammatory lesion groups (all *p* > 0.05). We also did not find significant between-group differences in all D* and *f* histogram metrics (all *p* > 0.05). Representative cases of lung cancer and inflammatory lesions are shown in **Figures 1**, **2**, respectively.

**Table 4:** Between-group differences in histogram parameters.

Parameter	Lung cancer (n = 39)	Inflammatory lesions (n = 20)	*p* value
*ADC histogram*			
Mean (×10^-3^ mm^2^/s)	1.416 ± 0.272	1.587 ± 0.250	0.022
Median (×10^-3^ mm^2^/s)	1.402 ± 0.264	1.579 ± 0.254	0.017
SD (×10^-3^ mm^2^/s)	0.241 ± 0.084	0.264 ± 0.075	0.302
Skewness	0.232 (0.094, 0.530)	-0.118 (-0.457, 0.339)	0.026
Kurtosis	2.993 ± 0.367	3.144 ± 0.509	0.195
*D histogram*			
Mean (×10^-3^ mm^2^/s)	0.990 (0.894, 1.193)	1.151 (1.050, 1.268)	0.021
Median (×10^-3^ mm^2^/s)	1.001 (0.916, 1.159)	1.243 (1.125, 1.348)	0.001
SD (×10^-3^ mm^2^/s)	0.418 (0.261, 0.547)	0.381 (0.266, 0.537)	0.974
Skewness	0.294 (-0.001, 0.436)	0.080 (-0.145, 0.288)	0.053
Kurtosis	3.249 ± 1.034	3.048 ± 0.808	0.452
D* histogram			
Mean (×10^-3^ mm^2^/s)	41.433 ± 15.895	43.806 ± 17.807	0.604
Median (×10^-3^ mm^2^/s)	13.538 (9.113, 25.339)	14.713 (9.968, 17.970)	0.873
SD (×10^-3^ mm^2^/s)	53.670 (44.784, 59.286)	55.318 (53.064, 57.793)	0.423
Skewness	1.268 ± 0.644	1.335 ± 0.886	0.740
Kurtosis	2.822 (2.125, 4.197)	2.457 (1.873, 2.769)	0.073
*f histogram*			
Mean (%)	26.190 (12.893, 34.909)	20.275 (13.792, 28.146)	0.554
Median (%)	19.878 (11.915, 32.691)	16.212 (11.326, 28.087)	0.387
SD (%)	19.358 (10.533, 25.203)	18.642 (14.426, 26.733)	0.642
Skewness	0.374 ± 0.525	0.637 ± 0.608	0.089
Kurtosis	2.455 (1.842, 2.627)	2.349 (1.886, 2.984)	0.987
*K_app_ histogram*			
Mean	0.726 ± 0.132	0.652 ± 0.100	0.030
Median	0.739 ± 0.125	0.658 ± 0.106	0.017
SD	0.223 (0.153, 0.302)	0.160 (0.133, 0.199)	0.007
Skewness	-0.947 (-1.236, 0.191)	-0.498 (-0.670, 0.294)	0.113
Kurtosis	4.865 (4.057, 5.676)	3.848 (3.213, 4.493)	0.008
*D_app_ histogram*			
Mean (×10^-3^ mm^2^/s)	1.727 (1.385, 2.414)	1.886 (1.383, 2.254)	0.994
Median (×10^-3^ mm^2^/s)	1.712 (1.284, 2.338)	1.795 (1.304, 2.196)	0.873
SD (×10^-3^ mm^2^/s)	0.499 (0.377, 0.623)	0.492 (0.366, 0.622)	0.904
Skewness	0.417 ± 0.391	0.027 ± 0.723	0.009
Kurtosis	2.843 (2.527, 3.449)	2.806 (2.379, 3.220)	0.665

Normally distributed data are expressed as mean ± SD and compared using two-sample t-test, while non-normally distributed data are expressed as median (lower quartile, upper quartile) and compared using Mann-Whitney U-test. SD, standard deviation.

### Independent predictors of lung cancer

As shown in **Table 5**, univariate logistic regression analysis revealed that ADC^mean^, ADC^median^, ADC^skewness^, *D* ^median^, K_app_^mean^, K_app_^median^, K_app_^SD^, K_app_^kurtosis^ and D_app_^skewness^ were statistically significant variables in the evaluation of lung cancer (all *p* < 0.05). Further multivariate logistic regression analysis showed that ADC^skewness^ (odds ratio [OR] = 7.061, *p* = 0.019) and *D* ^median^ (OR = 0.044, *p* = 0.031) were independent predictors of lung cancer.

**Table 5:** Results of univariate and multivariate logistic regression analyses.

	Univariate analysis		Multivariate analysis	
	OR (95% CI)	*p* value	OR (95% CI)	*p* value
*ADC histogram*				
Mean (×10^-4^ mm^2^/s)	0.782 (0.626-0.977)	0.030		
Median (×10^-4^ mm^2^/s)	0.771 (0.616-0.967)	0.024		
Skewness	8.212 (1.589-42.449)	0.012	7.061 (1.385-35.990)	0.019
*D histogram*				
Mean (×10^-3^ mm^2^/s)	0.063 (0.004-1.104)	0.059		
Median (×10^-3^ mm^2^/s)	0.035 (0.002-0.571)	0.019	0.044 (0.003-0.756)	0.031
*K_app_ histogram*				
Mean (×10^-1^)	1.696 (1.035-2.781)	0.036		
Median (×10^-1^)	1.835 (1.088-3.096)	0.023		
SD (×10^-1^)	2.897 (1.226-6.844)	0.015		
Kurtosis	1.603 (1.048-2.451)	0.029		
*D_app_ histogram*				
Skewness	4.213 (1.272-13.954)	0.019		

Variables with p < 0.05 in the univariate logistic regression analysis were included in the multivariate logistic regression analysis. OR, odds ratio; CI, confidence interval.

### Diagnostic performance of whole-lesion histogram metrics

**Figure 3:**
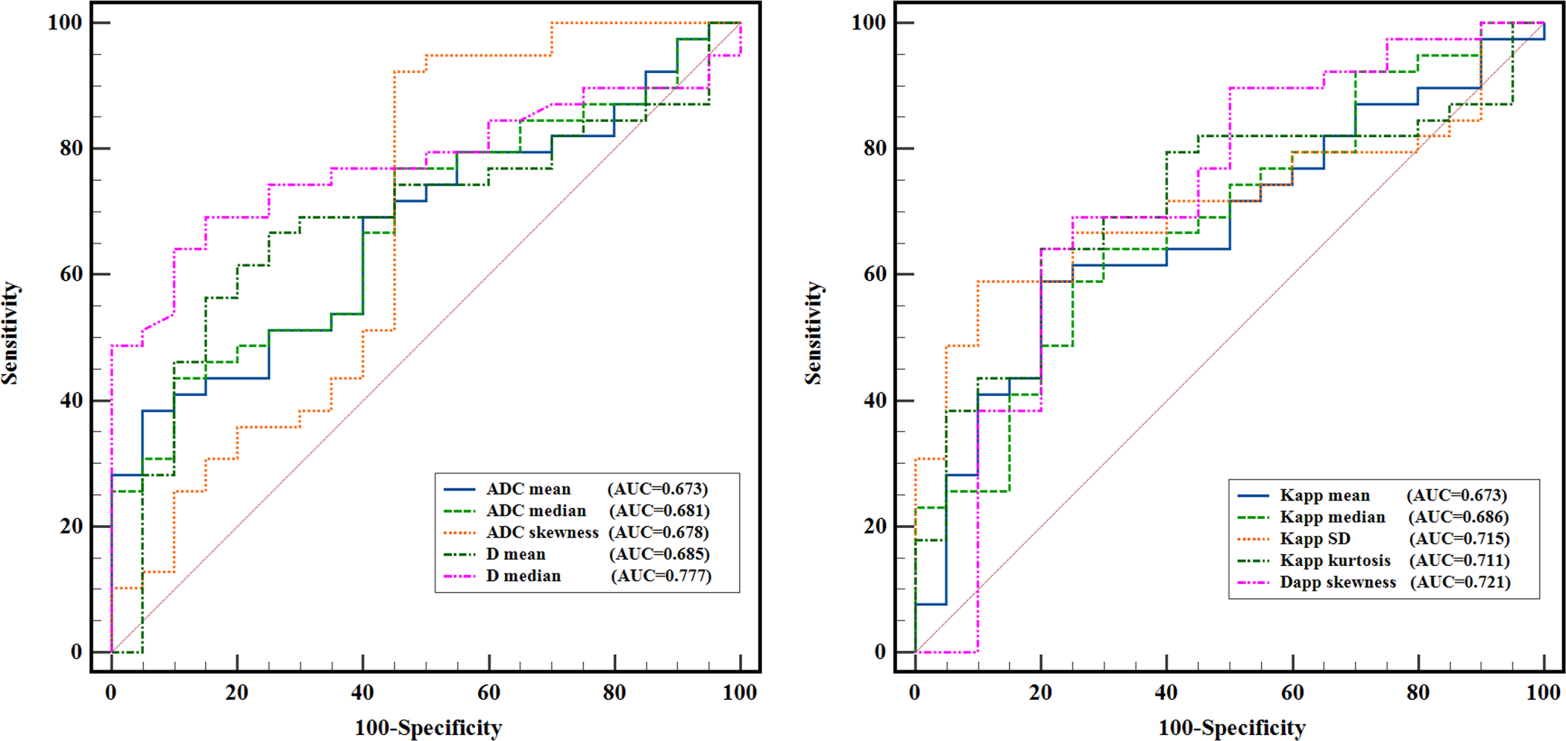
ROC curves of the quantitative histogram metrics for differentiating lung cancer from inflammatory lesions.

**Figure 3** shows the ROC curves of different histogram parameters for differentiating malignant lung cancers from benign inflammatory lesions, and the measurements of ROC analyses are shown in **Table 6**. ROC curve analysis revealed that *D* ^median^ showed the best performance for differentiating lung cancer from inflammatory lesions, with an area under the ROC curve of 0.777 (95% confidence interval [CI]: 0.660-0.984). Using a *D* ^median^ of 1.091 × 10^-3^ mm^2^/s as the optimal cut-off value, the sensitivity, specificity, PPV and NPV were 69.23%, 85.00%, 90.00% and 58.62%, respectively. The AUC values of ADC^mean^, ADC^median^ and ADC^skewness^, *D* ^mean^, K_app_^mean^, K_app_^median^, K_app_^SD^, K_app_^kurtosis^ and D_app_^skewness^ were 0.673 (95% CI: 0.536-0.810), 0.681 (95% CI: 0.544-0.818), 0.678 (95% CI: 0.517-0.840), 0.685 (95% CI: 0.543-0.826), 0.673 (95% CI: 0.532-0.814), 0.686 (95% CI: 0.546-0.826), 0.715 (95% CI: 0.586-0.845), 0.711 (95% CI: 0.577-0.846), and 0.721 (95% CI: 0.571-0.870), respectively. Further pairwise comparisons of ROC curves did not yield significant differences in AUCs (all *p* > 0.05). ADC^skewness^ had the highest sensitivity (92.31%) and ADC^mean^ showed the highest specificity (95.00%) in differentiating lung cancer from inflammatory lesions.

**Table 6:** ROC analysis results of whole-lesion histogram parameters for distinguishing lung cancer from benign lung lesions.

Parameter	*p* value	AUC (95% CI)	OCVs	Sensitivity (%)	Specificity (%)	PPV (%)	NPV (%)
ADC^mean^ (×10^-3^ mm^2^/s)	0.022	0.673 (0.536-0.810)	< 1.255	38.46	95.00	93.75	44.19
ADC^median^ (×10^-3^ mm^2^/s)	0.017	0.681 (0.544-0.818)	< 1.339	43.59	90.00	89.47	45.00
ADC^skewness^	0.026	0.678 (0.517-0.840)	> -0.095	92.31	55.00	80.00	78.57
*D* ^mean^ (×10^-3^ mm^2^/s)	0.021	0.685 (0.543-0.826)	< 1.057	66.67	75.00	86.67	55.17
*D* ^median^ (×10^-3^ mm^2^/s)	0.001	0.777 (0.660-0.894)	< 1.091	69.23	85.00	90.00	58.62
K_app_^mean^	0.030	0.673 (0.532-0.814)	> 0.701	58.97	80.00	85.19	50.00
K_app_^median^	0.017	0.686 (0.546-0.826)	> 0.692	64.10	70.00	80.65	50.00
K_app_^SD^	0.007	0.715 (0.586-0.845)	> 0.208	58.97	90.00	92.00	52.94
K_app_^kurtosis^	0.008	0.711 (0.577-0.846)	> 4.522	64.10	80.00	86.21	53.33
D_app_^skewness^	0.009	0.721 (0.571-0.870)	> 0.251	69.23	75.00	84.38	55.56

ROC, receiver operating characteristic; AUC, area under the curve; OCVs, optimal cut-off values; CI, confidence interval; PPV, positive predictive value; NPV, negative predictive value.

## Discussion

Using whole-lesion volume histogram analysis, the present study suggests that histogram metrics of DWI, IVIM and DKI parameters can objectively provide the overall histologic information of lung lesions and are clinically feasible and of value in differentiating lung cancer from benign inflammatory lesions. Our study also found significant between-group differences in histogram features of multiple magnetic resonance diffusion parameters, indicating that lung cancer has different histological features compared with inflammatory lesions. Furthermore, of all the histogram metrics, *D* ^median^ showed the best diagnostic performance in differentiating lung cancer from benign inflammatory lesions, and ADC^skewness^ and *D* ^median^ were identified as independent predictors of lung cancer. Our findings confirmed the potential value of histogram analysis of quantitative MRI parameters in the diagnosis of lung lesions.

An important finding of our study was that lung cancer showed lower ADC^mean^, ADC^median^, *D* ^mean^ and *D* ^median^ values compared to inflammatory lesions. Both the ADC and *D* are diffusion-related parameters, and prior study has demonstrated a strong negative association between tumor cellularity and the ADC value (16). Thus, given that malignant tumors usually have rapid cell proliferation and high cell density and malignant tumor cells have large nuclei and little cytoplasm, lower ADC and *D* histogram metrics (mean and median) in lung cancer may be attributed to higher cellularity and reduced extracellular space that leads to a more pronounced restriction of diffusional motion of water molecules. Using single-section ROI method and whole-lesion histogram analysis, prior studies have consistently demonstrated significantly lower ADC^mean^ and *D* ^mean^ values in malignant lung tumors than benign lesions (3, 6, 7, 9, 13, 17–19). In contrast, another study based on whole-lesion histogram analysis revealed significantly higher ADC^mean^ and ADC^median^ values in lung cancer compared with benign inflammatory lesions (20). This opposite result may be attributed to the selection of different benign lung lesions, as they included lung cancer and specific infectious lesions (pulmonary abscesses and mycobacterium infections) for comparison. Overall, our findings of reduced ADC^mean^ and ADC^median^ in lung cancer relative to inflammatory lesions are compatible with these previous studies.

Another important finding of our study was that lung cancer had significantly higher values in most of the K_app_ histogram metrics (mean, median, SD and kurtosis) compared to inflammatory lesions. DKI detects non-Gaussian interactions of water molecules within tissue environments, and the additional parameter derived from DKI model, K_app_, may reflect the heterogeneity and irregularity of cellular microstructure, as well as the amounts of interfaces within cellular tissues (5). Thus, the possible reason for the higher histogram metrics of DKI parameters in lung cancer is that the cellular structure and tissue environment of lung cancer are more complex than those of inflammatory lesions, leading to more significant non-Gaussian distribution in lung cancer. Similarly, higher mean K_app_ value in malignant pulmonary tumors than in benign lesions has been demonstrated by previous studies using single-section ROI analyses (3, 21). However, another study found that the K_app_ of malignant tumors was lower than that of benign lesions, although the difference was not statistically significant (7). Inconsistency of previous research results may be associated with differences in the selection of *b*-values, the inclusion criteria of lung lesions and the chosen of analytical methods. Notably, most of previous studies only used single-slice ROI method to compare differences in DKI parameters between malignant and benign lung lesions, and did not consider the important aspects of texture and heterogeneity in the whole lesions. Considering that whole-lesion histogram analysis allows for a more comprehensive, intuitive and accurate assessment of the microscopic motion of water molecules in tissues compared to traditional single-section ROI method, our findings may provide more reliable biomarkers for the differential diagnosis of lung lesions.

Additionally, we found that both the ADC^skewness^ and D_app_^skewness^ values of lung cancer showed a “positive skewness” pattern and were significantly higher than that of inflammatory lesions, which is consistent with a recent study (13). Skewness reflects the degree of asymmetry of the data, and the skewness of the normal distribution is 0. A “positive skewness” pattern of the ADC and D_app_ means that more data are distributed to the left of the mean value. Higher ADC^skewness^ and D_app_^skewness^ values in lung cancer might be due to its higher cell density and more pixels had lower ADC and D_app_ values.

Both the quantitative parameters D* and *f* derived from IVIM are related to perfusion. In IVIM theory, D* is considered to be proportional to the blood velocity and capillary segment length, while *f* reflects the blood perfusion of the tissue and is related to blood volume (4, 22). Differences in D* and *f* between malignant and benign lung lesions have been inconsistent in previous studies using single-section ROI analyses. Some studies found no significant differences in both D* and *f* values between lung cancer and benign lesions (7, 23, 24), while others revealed significant higher D* or lower *f* values in lung cancer compared to benign lesions (6, 25, 26). For whole-lesion histogram features, in line with all prior whole-lesion histogram studies (9, 13), we also did not find significant differences in any histogram metrics of D* and *f* between lung cancer and benign inflammatory lesions, suggesting that D* and *f* histogram characteristics cannot reflect the characteristics of lung lesions well and have relatively limited value in the differential diagnosis of pulmonary lesions. Two possible reasons may account for the non-significant differences in D* and *f* between lung cancer and inflammatory lesions. First, both our findings and those of previous studies revealed that measurement consistency of D* and *f* among different observers were relatively low (3, 13), and prior studies also found that D* had a relatively large SD (23, 26), indicating poor reproducibility of these two parameters. Second, malignant lung tumors have rich neovascularization due to the high expression of vascular endothelial growth factor, which may theoretically increase blood supply of tumor tissues (27); however, malignant tumors tend to have immature new blood vessels with increased microvascular permeability and relatively insufficient veins or lymphatic vessels to drain the excess leaking fluid, which may result in compression of tumoral vessels and lead to reduced blood perfusion in the tissues. Thus, non-significant difference in D* and *f* values between lung cancer and benign lesions may be due to the combined effect of the two factors.

Further ROC analyses suggested that whole-lesion quantitative histogram analysis of conventional DWI, IVIM and DKI are clinically feasible and of potential value in differentiating lung cancer from inflammatory lesions. We found that ADC^skewness^ had the highest sensitivity and ADC^mean^ achieved the highest specificity in differentiating lung cancer from inflammatory lesions. This finding means that ADC^skewness^, a metric that reflects the higher cellular density in malignant tumors, has a relatively better performance in the diagnosis of lung cancer; while ADC^mean^, a metric that measures the overall diffusion and perfusion information of lesions, shows a better performance in identifying inflammatory lesions. Moreover, our study revealed that *D* ^median^ had the highest AUC in differentiating lung cancer from inflammatory lesions, and further multivariate logistic regression analysis found that the *D* ^median^ was an independent predictor of lung cancer. These findings suggest that *D* ^median^ may act as a reliable biomarker for differentiating lung cancer from inflammatory lesions, and highlight the superiority of the whole-lesion volume histogram analysis in the differential diagnosis of solitary lung lesions. A recent meta-analysis also confirmed that *D* had the best performance in the differential diagnosis between malignant and benign solitary pulmonary lesions (28). Unlike ADC that is sensitive to both the diffusion of water molecules and microcirculation perfusion, *D* reflects only the pure diffusion of water molecules by removing the effect of perfusion portion and can achieve a more accurate assessment of the restricted movements of water molecules in tissues. Malignant tumors have high cell density and reduced extracellular space; thus, the low diffusion coefficient detected in lung cancer may be mainly related to pure molecular diffusion rather than perfusion.

This study has several limitations. First, the sample size included was small, resulting in a small number of lung lesions available for analysis. We also did not evaluate the diagnostic performance of whole-lesion histogram analysis of DWI, IVIM and DKI in distinguishing subtypes of lung cancer due to a small sample size. Future studies with larger sample sizes are needed to verify our findings and to further assess the role of DWI, IVIM and DKI histogram features in the differential diagnosis of pathological types of lung cancer. Second, we only included lung lesions with a maximum diameter ≥ 15 mm for histogram analysis, which may lead to selection bias. Third, some inflammatory lesions were confirmed based on long-term follow-up rather than by pathological examinations, which may lead to inaccurate results to some extent.

In summary, the results of our study suggest that whole-lesion quantitative histogram analysis of DWI, IVIM and DKI parameters provides a useful tool for differentiating lung cancer from inflammatory lesions, and *D* ^median^ may act as a potential biomarker for the differential diagnosis of solitary pulmonary lesions.

## Data availability statement

The original contributions presented in the study are included in the article/supplementary material. Further inquiries can be directed to the corresponding authors.

## Ethics statement

The studies involving human participants were reviewed and approved by the Institutional Review Board of The First Affiliated Hospital of Henan University. The patients/participants provided their written informed consent to participate in this study.

## Author contributions

JZ, ZW, JL and BW contributed to the conception and design of the study. JL, BW, ZH, YZ, SZ, SG, SX, XW and TT contributed to literature search, data analysis and data interpretation. JL and BW contributed to the drafting of the manuscript. JZ, ZW and JL obtained funding to support this work. JZ and BW critically revised the manuscript. All authors approved the final version of the manuscript.

## References

[1] Xia C Dong X Li H Cao M Sun D He S. Cancer statistics in China and united states 2022: profiles, trends, and determinants. Chin Med J (Engl). (2022) 135(5):584–90. doi: 10.1097/cm9.0000000000002108 PMC8920425 35143424

[2] Shen G Jia Z Deng H. Apparent diffusion coefficient values of diffusion-weighted imaging for distinguishing focal pulmonary lesions and characterizing the subtype of lung cancer: a meta-analysis. Eur Radiol (2016) 26(2):556–66. doi: 10.1007/s00330-015-3840-y 26003791

[3] Zheng Y Li J Chen K Zhang X Sun H Li S. Comparison of conventional DWI, intravoxel incoherent motion imaging, and diffusion kurtosis imaging in differentiating lung lesions. Front Oncol (2021) 11:815967. doi: 10.3389/fonc.2021.815967 35127530 PMC8810497

[4] Le Bihan D Breton E Lallemand D Aubin ML Vignaud J Laval-Jeantet M. Separation of diffusion and perfusion in intravoxel incoherent motion MR imaging. Radiology (1988) 168(2):497–505. doi: 10.1148/radiology.168.2.3393671 3393671

[5] Rosenkrantz AB Padhani AR Chenevert TL Koh DM De Keyzer F Taouli B. Body diffusion kurtosis imaging: Basic principles, applications, and considerations for clinical practice. J Magn Reson Imaging (2015) 42(5):1190–202. doi: 10.1002/jmri.24985 26119267

[6] Deng Y Li X Lei Y Liang C Liu Z. Use of diffusion-weighted magnetic resonance imaging to distinguish between lung cancer and focal inflammatory lesions: A comparison of intravoxel incoherent motion derived parameters and apparent diffusion coefficient. Acta Radiol (2016) 57(11):1310–7. doi: 10.1177/0284185115586091 25972370

[7] Wan Q Deng YS Lei Q Bao YY Wang YZ Zhou JX. Differentiating between malignant and benign solid solitary pulmonary lesions: Are intravoxel incoherent motion and diffusion kurtosis imaging superior to conventional diffusion-weighted imaging? Eur Radiol (2019) 29(3):1607–15. doi: 10.1007/s00330-018-5714-6 30255258

[8] Nougaret S Vargas HA Lakhman Y Sudre R Do RK Bibeau F. Intravoxel incoherent motion-derived histogram metrics for assessment of response after combined chemotherapy and radiation therapy in rectal cancer: Initial experience and comparison between single-section and volumetric analyses. Radiology (2016) 280(2):446–54. doi: 10.1148/radiol.2016150702 PMC4976464 26919562

[9] Yuan M Zhong Y Zhang YD Yu TF Li H Wu JF. Volumetric analysis of intravoxel incoherent motion imaging for assessment of solitary pulmonary lesions. Acta Radiol (2017) 58(12):1448–56. doi: 10.1177/0284185117698863 28269992

[10] Peng Y Tang H Meng X Shen Y Hu D Kamel I. Histological grades of rectal cancer: whole-volume histogram analysis of apparent diffusion coefficient based on reduced field-of-view diffusion-weighted imaging. Quant. Imaging Med Surg (2020) 10(1):243–56. doi: 10.21037/qims.2019.11.17 PMC6960428 31956546

[11] Zhang Y Chen J Liu S Shi H Guan W Ji C. Assessment of histological differentiation in gastric cancers using whole-volume histogram analysis of apparent diffusion coefficient maps. J Magn Reson Imaging (2017) 45(2):440–9. doi: 10.1002/jmri.25360 27367863

[12] Li T Hong Y Kong D Li K. Histogram analysis of diffusion kurtosis imaging based on whole-volume images of breast lesions. J Magn Reson Imaging (2020) 51(2):627–34. doi: 10.1002/jmri.26884 31385429

[13] Zhu Q Ren C Xu JJ Li MJ Yuan HS Wang XH. Whole-lesion histogram analysis of mono-exponential and bi-exponential diffusion-weighted imaging in differentiating lung cancer from benign pulmonary lesions using 3 T MRI. Clin Radiol (2021) 76(11):846–53. doi: 10.1016/j.crad.2021.07.003 34376284

[14] DeLong ER DeLong DM Clarke-Pearson DL. Comparing the areas under two or more correlated receiver operating characteristic curves: a nonparametric approach. Biometrics (1988) 44(3):837–45. doi: 10.2307/2531595 3203132

[15] Genovese CR Lazar NA Nichols T. Thresholding of statistical maps in functional neuroimaging using the false discovery rate. Neuroimage (2002) 15(4):870–8. doi: 10.1006/nimg.2001.1037 11906227

[16] Yin Y Sedlaczek O Muller B Warth A Gonzalez-Vallinas M Lahrmann B. Tumor cell load and heterogeneity estimation from diffusion-weighted MRI calibrated with histological data: An example from lung cancer. IEEE Trans Med Imaging (2018) 37(1):35–46. doi: 10.1109/tmi.2017.2698525 28463188

[17] Çakır Ç. Gençhellaç H Temizöz O Polat A Şengül E Duygulu G. Diffusion weighted magnetic resonance imaging for the characterization of solitary pulmonary lesions. Balkan. Med J (2015) 32(4):403–9. doi: 10.5152/balkanmedj.2015.15663 PMC4692341 26740901

[18] Çakmak V Ufuk F Karabulut N. Diffusion-weighted MRI of pulmonary lesions: Comparison of apparent diffusion coefficient and lesion-to-spinal cord signal intensity ratio in lesion characterization. J Magn Reson Imaging (2017) 45(3):845–54. doi: 10.1002/jmri.25426 27519160

[19] Liang J Li J Li Z Meng T Chen J Ma W. Differentiating the lung lesions using intravoxel incoherent motion diffusion-weighted imaging: a meta-analysis. BMC Cancer (2020) 20(1):799. doi: 10.1186/s12885-020-07308-z 32831052 PMC7446186

[20] Usuda K Iwai S Yamagata A Iijima Y Motono N Matoba M. Whole-lesion apparent diffusion coefficient histogram analysis: Significance for discriminating lung cancer from pulmonary abscess and mycobacterial infection. Cancers (Basel). (2021) 13(11):2720. doi: 10.3390/cancers13112720 34072867 PMC8198705

[21] Das SK Yang DJ Wang JL Zhang C Yang HF. Non-Gaussian diffusion imaging for malignant and benign pulmonary nodule differentiation: a preliminary study. Acta Radiol (2017) 58(1):19–26. doi: 10.1177/0284185116639763 27055919

[22] Le Bihan D. Intravoxel incoherent motion perfusion MR imaging: A wake-up call. Radiology (2008) 249(3):748–52. doi: 10.1148/radiol.2493081301 19011179

[23] Yuan M Zhang YD Zhu C Yu TF Shi HB Shi ZF. Comparison of intravoxel incoherent motion diffusion-weighted MR imaging with dynamic contrast-enhanced MRI for differentiating lung cancer from benign solitary pulmonary lesions. J Magn Reson Imaging (2016) 43(3):669–79. doi: 10.1002/jmri.25018 26340144

[24] Wan Q Deng YS Zhou JX Yu YD Bao YY Lei Q. Intravoxel incoherent motion diffusion-weighted MR imaging in assessing and characterizing solitary pulmonary lesions. Sci Rep (2017) 7:43257. doi: 10.1038/srep43257 28225064 PMC5320549

[25] Wang LL Lin J Liu K Chen CZ Liu H Lv P. Intravoxel incoherent motion diffusion-weighted MR imaging in differentiation of lung cancer from obstructive lung consolidation: comparison and correlation with pharmacokinetic analysis from dynamic contrast-enhanced MR imaging. Eur Radiol (2014) 24(8):1914–22. doi: 10.1007/s00330-014-3176-z 24788038

[26] Zhou SC Wang YJ Ai T Huang L Zhu TT Zhu WZ. Diagnosis of solitary pulmonary lesions with intravoxel incoherent motion diffusion-weighted MRI and semi-quantitative dynamic contrast-enhanced MRI. Clin Radiol (2019) 74(5):409.e407–409.e416. doi: 10.1016/j.crad.2018.12.022 30795843

[27] Yi CA Lee KS Kim EA Han J Kim H Kwon OJ. Solitary pulmonary nodules: dynamic enhanced multi-detector row CT study and comparison with vascular endothelial growth factor and microvessel density. Radiology (2004) 233(1):191–9. doi: 10.1148/radiol.2331031535 15304661

[28] Chen Y Han Q Huang Z Lyu M Ai Z Liang Y. Value of IVIM in differential diagnoses between benign and malignant solitary lung nodules and masses: A meta-analysis. Front Surg (2022) 9:817443. doi: 10.3389/fsurg.2022.817443 36017515 PMC9396547

